# Adoption, Acceptability, and Effectiveness of a Mobile Health App for Personalized Prostate Cancer Survivorship Care: Protocol for a Realist Case Study of the Ned App

**DOI:** 10.2196/resprot.8051

**Published:** 2017-10-12

**Authors:** Quynh Pham, Joseph A Cafazzo, Andrew Feifer

**Affiliations:** ^1^ Institute of Health Policy, Management and Evaluation Dalla Lana School of Public Health University of Toronto Toronto, ON Canada; ^2^ Centre for Global eHealth Innovation Techna Institute University Health Network Toronto, ON Canada; ^3^ Institute of Biomaterials and Biomedical Engineering Faculty of Medicine University of Toronto Toronto, ON Canada; ^4^ Carlo Fidani Regional Cancer Centre Credit Valley Hospital Trillium Health Partners Mississauga, ON Canada; ^5^ Department of Surgery Faculty of Medicine University of Toronto Toronto, ON Canada

**Keywords:** prostate-specific antigen, prostate cancer survivorship, prostate cancer, patient-centered care, mobile health, mHealth, telemedicine, mobile health app, realist evaluation, case study

## Abstract

**Background:**

By 2030, prostate cancer will be the most commonly diagnosed cancer in North America. To mitigate this impending challenge, comprehensive support mechanisms for disease- and treatment-specific changes in health and well-being must be proactively designed and thoughtfully implemented for streamlined survivorship care. mHealth apps have been lauded as a promising complement to current outpatient treatment and monitoring strategies, but have not yet been widely used to support prostate cancer survivorship needs. A realist evaluation is needed to examine the impact of such apps on the prostate cancer survivorship experience.

**Objective:**

We seek to gain an understanding of how an mHealth app for prostate cancer survivorship care called Ned (No Evident Disease) is adopted and accepted by patients, caregivers, and clinicians. We also aim to determine the effect of Ned on health-related quality of life, satisfaction with cancer care, unmet needs, self-efficacy, and prostate cancer-related levels of anxiety.

**Methods:**

The Ned case study is a 12-month mixed-methods embedded single-case study with a nested within-group pre-post comparison of health outcomes. We will give 400 patients, 200 caregivers, and 10 clinicians access to Ned. Participants will be asked to complete study assessments at baseline, 2 months, 6 months, and 12 months. We will conduct 30 semistructured qualitative interviews with patients (n=20) and their caregivers (n=10) poststudy to gain insight into their experience with the app.

**Results:**

We recruited our first survivor in October 2017 and anticipate completing this study by May 2019.

**Conclusions:**

This will, to our knowledge, be the first realist case study to evaluate an app for prostate cancer survivorship care. Prostate cancer survivors are set to increase in number and longevity, heightening the need for integrated survivorship solutions to provide them with optimal and durable outcomes. The knowledge gained from this study will comprehensively inform how and why Ned works, for whom, and in what circumstances. Understanding the impact of digital health interventions such as Ned on how survivors care for themselves is critical to realizing patient-centered care.

## Introduction

### Background

By 2030, prostate cancer will be the most commonly diagnosed cancer in North America [1,2]. The global population is also aging, with the number of those aged 60 years and over expected to rise from 1 in 10 currently to 1 in 6 in the next 10 years; the United Nations estimates that by 2050, this number will grow to be 1 in 3 [3]. Given the increased risk with age for this increasingly high-mortality cancer, an unprecedented population of prostate cancer survivors will require specialized support and services from a potentially underprepared health care system [4,5]. To mitigate these impending challenges, durable support mechanisms for disease- and treatment-specific changes in health and well-being must be proactively designed and thoughtfully implemented for streamlined survivorship care.

The definition of prostate cancer survivorship has changed with the understanding that the patient experience encompasses far more than just medical treatment [6]. Following diagnosis and primary treatment, patients are discharged into the community and face significant long-term health challenges as a result of their treatment; these include physical, rehabilitation-related, psychological, and emotional needs, coupled with the needs of their caregiver [7]. Seminal research on quality of life among prostate cancer survivors has revealed a decrease in quality of life across numerous domains, notably sexual and urinary function [8]. These physical aspects of prostate cancer survivorship are further exacerbated by anxiety in both patients and their caregivers as they follow their prostate-specific antigen (PSA) values to assess for recurrence [9]. These aspects should be systematically captured via patient-reported outcome measures (PROMs) to inform personalized, patient-centered, and value-based care [10]. Despite robust efforts, there has not yet been a successful initiative to link the collection of prostate cancer-specific PROMs with clinical markers such as PSA values in a way that facilitates and positively informs the clinical interaction; the combination of both measures may advance an evidenced-based understanding of both physiological and personal self-reported prostate cancer status.

Three critical problems have been identified in the overall clinical management of prostate cancer survivorship: (1) patients do not know how they are doing and how they compare with other matched patients; (2) clinicians are not optimally informed about patient issues in a systematic and evidenced-based manner; and (3) patients receive fragmented care [11]. There is also a lack of communication and information sharing between clinicians, patients, and caregivers, which adds further strain on survivor supportive care needs [12-14].

#### Digitally Mediated Prostate Cancer Survivorship Care

In recent years, digital health interventions have presented new opportunities and challenges for cancer care, with systematic reviews suggesting their overall usefulness and acceptance for cancer patients and their caregivers [15]. Research on how prostate cancer survivors understand the health information presented to them and communicate that information to their circle of care suggests that they are willing and able to use digital health interventions for illness management support [16]. Prostate and testicular cancer survivors in particular have a more positive attitude toward online contact with clinicians than do survivors of other cancer groups [17]. However, these survivors also have a greater need for decision-making support and preparation before communicating with health personnel [16]. Successes in remote and digital prostate cancer survivorship care delivery have been realized, with recent work on the effectiveness of urology telemedicine clinics suggesting that the remote delivery of general urologic care is cost effective and results in high patient satisfaction [18]. Efforts have also been made to incorporate electronic PROMs into the management of survivorship care for improved communication between patients and their care team [19]. This has been complemented by work in Web-based education programs for prostate cancer survivors transitioning from active treatment [20], as well as mobile phone-based interventions to increase adherence to oral antiprostate cancer medications [21]. Further, the collection of PROMs and their linkage with clinical prostate cancer registry data has been operationalized [22]. However, formal evaluations have not yet been conducted to examine the impact of these systems, leaving an unfinished translation from theoretical framework to practical implementation.

#### Ned: A Prostate Cancer Survivorship App

The recent endorsement of mHealth, broadly defined as a health intervention that has been operationalized into an app for patient use and is delivered or supported through the use of wireless devices (eg, mobile phones, tablets, media players, wearables), has been lauded as a promising complement to current outpatient treatment and monitoring strategies for chronic care [23]. In particular, the ubiquity of mobile phone use has facilitated the adoption of mHealth apps for immunization record keeping [24], heart health [25], and diabetes self-management [26]. In November 2012, a proof-of-concept mHealth app prototype for personalized prostate cancer survivorship care was conceptualized at the Hacking Health Codeathon in Toronto, Canada [27]. From 2014 to 2017, the app was translated from prototype to product, and a user-centered needs assessment and usability evaluation were conducted to inform app content and functionality (University Health Network Research Ethics Board [REB] ID#14-7510). Ned (No Evident Disease) is the first app to provide patients with access to individual-level PSA values streamed directly from the Ontario Laboratories Information System (OLIS) to their own smartphone. The mHealth app for patients and their caregivers, along with a clinician-facing app and complementary Web-based dashboard, were developed using Fast Healthcare Interoperability Resources, which facilitate interoperability with existing Canadian provincial and federal health care assets [28]. Ned aims to promote self-care by informing patients directly of their PSA results and providing them with a personalized view of their own symptoms. It supports real-time clinical decision making by providing clinicians with PROMs collected in-app, and includes a curated educational feed and support group links. Ned is not meant to take the place of an informative discussion between patient and provider; however, there is an acknowledgement that, in reality, patient-provider interactions may be brief, and patients may require additional communication channels, which may lead to more meaningful interactions and improve shared decision making.

#### Innovative mHealth Clinical Trials

Previous work by our research group has identified a homogeneity in the range of study designs used to evaluate mHealth apps [29]. mHealth researchers have been reluctant to deviate from traditional study designs, namely the parallel-group randomized controlled trial. We believe there is value in diversifying the types of study designs used in the mHealth field—it is imperative that we broaden the range of research questions being asked to elicit useful, relevant, and timely research findings that keep pace with the technology under study [30-32]. We have therefore aimed to design an evaluation that asks not “does Ned work,” but instead asks “how or why does Ned work or not work, for whom, to what extent, in what respects, in what circumstances, and over what duration?” [33]. We posit that this paradigm shift in evaluative approach will capture the nuanced stories of how Ned is received by its intended users and inform meaningful iterations of the app to optimize prostate cancer survivorship care.

### Objectives

This protocol outlines a pragmatic, mixed-methods embedded single-case study with a nested within-group pre-post comparison of health outcomes, guided by Pawson and Tilley’s realist evaluation principles [33], to elicit a context-focused and mechanism-driven understanding of outcomes derived from the use of Ned by patients, caregivers, and clinicians within a public hospital network in Toronto, Canada. The aims of this study are 2-fold: (1) to gain an understanding of how a prostate cancer survivorship app called Ned for viewing laboratory results and collecting patient-reported outcomes is adopted and accepted by patients, caregivers, and clinicians; and (2) to determine the effect of Ned on health-related quality of life, satisfaction with cancer care, unmet needs, self-efficacy, and prostate cancer-related levels of anxiety.

## Methods

### Theoretical Approach

We will use the realist evaluation framework to guide the interpretation of this case study [33,34]. Realist evaluations are designed to inform an understanding of how and why interventions work or do not work in particular contexts. They belong to a family of theory-based evaluation approaches that aim to establish the “program theory” of an intervention: the mechanisms that are likely to operate, the contexts in which they might operate, and the outcomes that will be observed if they operate as expected [35]. Realist approaches assume that nothing works everywhere for everyone, and that it is the context in which an intervention operates that will significantly affect outcomes. Researchers who engage with this methodology collect data on the contextual features that might affect how and for whom an intervention is expected to work, and then analyze the data to examine the interaction between context and mechanisms of action [36]. The adoption of this methodology has grown in the health services research community and is now recognized as a powerful approach to designing and analyzing complex evaluations [37]. Realist evaluations are particularly well suited for evaluating digital health interventions given the complex sociotechnical relationship between users and technology [38]. They are also more conceptually suited to capturing the dependency between a technology’s success and its implementation plan [36]. We believe this approach will enable us to draw meaningful insights from our case study that are representative of how Ned performs across various use cases and stakeholder groups.

### Trial Design

The Ned case study is a 12-month mixed-methods embedded single-case study with a nested within-group pre-post comparison of health outcomes. We will evaluate the adoption, acceptability, and effectiveness of Ned as perceived by the 400 patients, 200 caregivers, and 10 clinicians who are given access to the app. Our aims are both observational and experimental: we are interested in deriving a deep understanding of how Ned affects survivorship care, but also in establishing parameters of possible clinical change to inform the effect of the app on quality-of-life outcomes. We also seek to identify the process mechanisms required to introduce Ned into a public health network, specifically the resources required to support the platform. This knowledge will inform future innovation diffusion efforts for Ned as we look to scale the platform across Canada. We will employ a combination of semistructured interviews, questionnaires, and qualitative observations to illustrate a rich picture of how Ned is adopted, whether it is accepted, and the effect it has on how prostate cancer survivors and their care team experience survivorship care. We have obtained REB approval from the Trillium Health Partners (THP) hospital network for this research (THP REB ID#826). Table 1 outlines the multiple embedded units of analyses planned for this study.

**Table 1:** Summary of embedded units of analyses for the evaluation of the Ned app.

Embedded unit of analysis	Data sources
Patient, caregiver, and clinician adoption of Ned	Analytics log data on the number of patients, caregivers, and clinicians who are invited to open a Ned account, and the consequent number of Ned accounts created
Patient acceptance of Ned	Analytics log data on the frequency, duration, depth, and breadth of patient engagement with the app
	Interviews with 20 patients poststudy
	Web-based questionnaire assessing acceptability
	Qualitative observation of patients using Ned to access prostate-specific antigen results and submit patient-reported outcome measures
Caregiver acceptance of Ned	Analytics log data on the frequency, duration, depth, and breadth of caregiver engagement with the app
	Interviews with 10 caregivers poststudy
Clinician acceptance of Ned	Analytics log data on the frequency, duration, depth, and breadth of clinician engagement with the app
	Wed-based questionnaire assessing system use to be completed by 10 clinicians
Clinical effectiveness of Ned	Five validated patient-reported outcome measures (ie, quality of life, treatment satisfaction, unmet needs, self-efficacy, anxiety) administered at baseline, 2 months, 6 months, and 12 months

### Eligibility Criteria

Patients must meet the following eligibility criteria to be enrolled into the study: (1) 18 years of age or older, (2) receiving care at the THP Mississauga Hospital or Credit Valley Hospital, (3) pathology report confirming prostate cancer diagnosis via transrectal, transperineal, or transurethral biopsy (standard 12-core template), (4) life expectancy more than 1 year, (5) no concomitant cancer diagnosis, (6) own a device that is compatible with the Ned app and is Web enabled through a data plan or Wi-Fi capabilities, or both (eg, laptop, desktop, tablet, smartphone), (7) able to read, write, and speak English.

Clinicians (ie, urologists, surgical oncologists, radiation oncologists) who are employed by the THP hospital group, practice at either the Mississauga Hospital or Credit Valley Hospital, and are involved in the care of patients with a diagnosis of prostate cancer are eligible to participate in this study; our research group has secured Privacy Impact Assessment and Threat Risk Assessment agreements with both hospitals. Caregivers must be paired with a patient who is enrolled in the study to be eligible for access to Ned. We broadly define caregivers here as a spouse, relative, friend, or formal caregiver of the patient who significantly contributes to their care.

### Recruitment

#### Clinician Recruitment

The clinical champion for Ned is a urologist at THP and will initiate clinician recruitment through snowball sampling his colleagues (clinicians from urology, urologic oncology, radiation oncology, and medical oncology departments) from both THP hospitals. Clinicians who express interest in joining the study will provide the clinical champion with their primary contact number. The clinical champion will forward this number to the study coordinator (SC), who will initiate first contact with the clinician through a telephone call. The SC will provide the clinician with information about the study procedures and set a time to meet with the clinician to install Ned in their clinic. The SC will collect informed consent directly from the clinician at their clinic before setting up their Ned account. Once the SC sets up the account, the clinician will enter their medical license number to receive PSA values from OLIS and release them to patients. Clinicians can then invite and add patients from their roster into the study.

#### Patient Recruitment

Patients will be recruited into the study through an invitation from their clinician during a visit to the clinic. If patients express interest in using Ned as part of their survivorship care resources and meet the study eligibility criteria as assessed by their clinician, their email address will be collected by the clinician and forwarded to the SC. The SC will then initiate first contact with the patient through email to confirm study eligibility, provide a study sheet with a brief description of the study (eg, study purpose and procedures, relevant risks and benefits), and obtain digital consent through a Web-based consent form that can be digitally signed by the patient. Once the patient’s digital consent is verified, they will be prompted to complete a series of demographic and baseline outcome questionnaires. The SC will receive a notification when the patient digitally consents to join the study and completes their baseline assessment, and will email the patient’s clinician to open a Ned account on the patient’s behalf. The clinician will log on to their own Ned account and invite their patient to use Ned. The patient will then have access to Ned and can invite and add their caregiver to use the app.

#### Caregiver Recruitment

**Figure 1:**
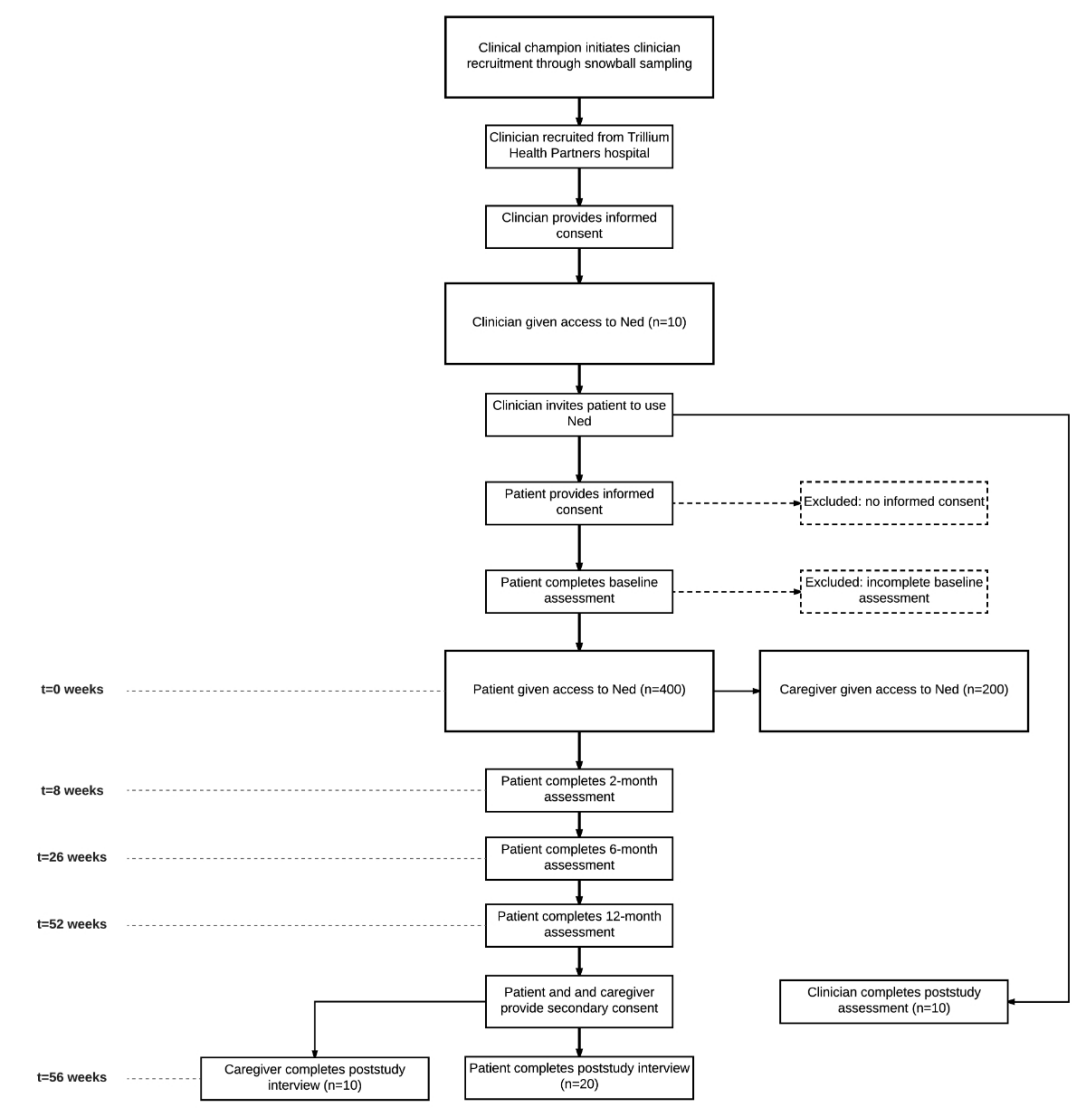
Ned study flowchart.

Caregivers will be invited to use Ned by their partners through a feature in the patient-facing version of Ned that allows them to add a caregiver to view their health data and complete PROMs on their behalf. When the patient uses this feature, the caregiver will receive an email with a link to sign up for Ned. The patient will verbally communicate a secret word to their caregiver, which the caregiver will then enter as part of the account creation process. The caregiver will then have access to Ned. Figure 1 presents the study flow for patients, caregivers, and clinicians from recruitment through to study conclusion.

### Consent

Clinicians will consent to participate in the Ned study by digitally signing a Web-hosted consent form provided to them by the SC in clinic. Patients will provide electronic consent by digitally signing a Web-hosted consent form provided to them by the SC through email. Caregivers will be asked to provide digital consent only if they express interest in attending a poststudy interview to share their experience of using Ned. Given that the “Add a caregiver” feature of Ned is controlled by the patient and is a native feature of the app itself, we believe that it is appropriate for patients to consent on behalf of any caregiver they add to use the app. Patients are clearly informed in their consent form that they have the ability to add a caregiver to use Ned; if they consent to using the app for the purposes of this research, they are consenting to be given access to all the features within Ned. Caregivers will be eligible to attend a poststudy interview only if their partner (the patient) has completed an interview themselves.

At study conclusion, we will ask patients and caregivers whether they would like to attend a poststudy interview to share their experience of using Ned. We will then collect a separate consent form from interested patients and caregivers at the interview. Rolling recruitment of patients who have completed the study and their paired caregiver will be done until 20 patients and 10 caregivers have been interviewed.

### Cost and Reimbursement

Patients will receive a Can $5 gift card for every study assessment completed and an additional Can $5 for completing all 4 study assessments, for a total of Can $25 as compensation for their participation in this study. If patients and caregivers choose to attend the 30-minute semistructured interview poststudy with the SC, they will receive a reimbursement of Can $25 for the cost of their parking. Clinicians will not be compensated for participating in this study.

### Intervention

The Ned app facilitates prostate cancer survivorship self-care and ownership of personal health data, and enables survivors to share their care plan with their caregiver and clinician to streamline care. Once patients complete the initial download of the Ned app and create an account, they are able to access all of Ned’s features. Patients can check their PSA values from directly within the app once their clinician has approved and released them. All values are first sent from OLIS to the clinician on their version of the Ned app. The clinician must review and approve these values by physically clicking on a button that confirms their attestation to the validity and appropriateness of the value. Only after the clinician has approved and released the PSA value to their patient does it get sent to the patient, who can then view it on their version of the Ned app.

Patients will have scheduled monthly PROMs, specifically the Expanded Prostate Cancer Index Composite (EPIC-26) [39] and the Functional Assessment of Cancer Therapy-Prostate [40], to measure their prostate cancer-specific quality of life. Patients will be prompted to complete these PROMs in-app and submit them directly to be viewed by their clinician on the Ned clinician dashboard. Ned will provide immediate feedback to patients on their health and well-being status with the submission of every PROM.

**Figure 2:**
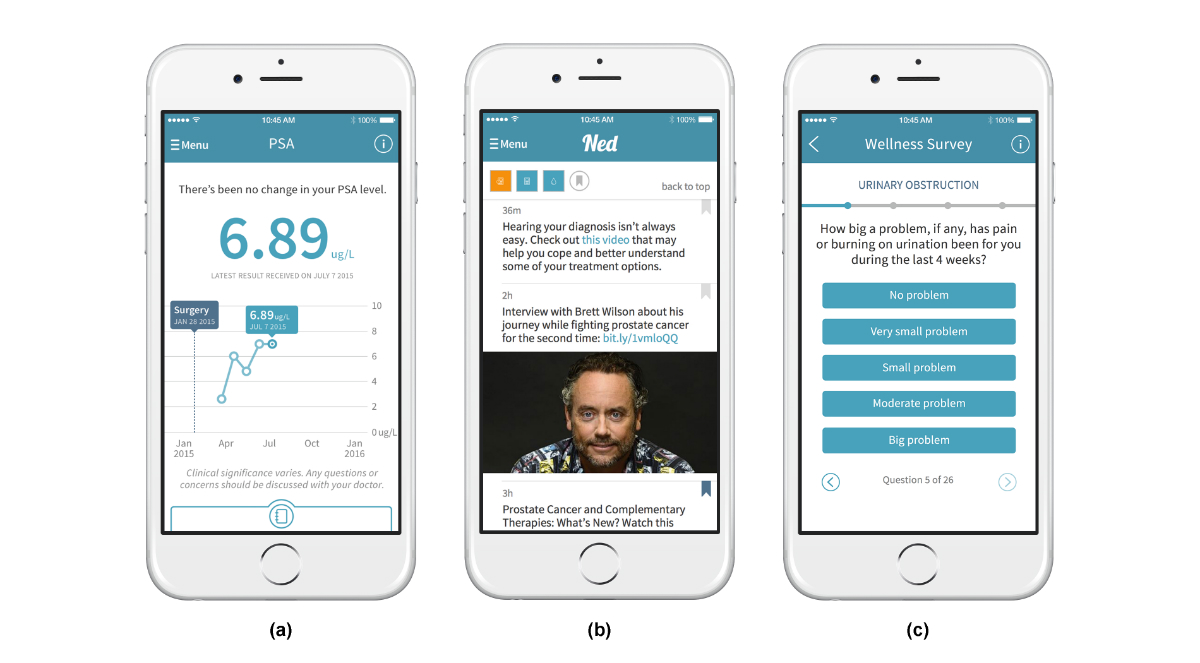
Screenshots of the Ned app. (a) Graphical view of prostate-specific antigen (PSA) results; (b) Ned’s Notes; and (c) the Expanded Prostate Cancer Index Composite (EPIC-26) survey.

The app also provides patients with access to Ned’s Notes, a curated feed of educational content coupled with upcoming local social events related to prostate cancer survivorship care. Patients are able to add caregivers to their account; when granted access, caregivers can complete PROMs on behalf of the patient, view PSA results, and take freehand notes on observable health concerns. The centralized hosting of laboratory and PROMs data enables both patients and clinicians to have access to shared data, in the hopes of fostering greater informed decision making. The Ned clinician app and dashboard will provide clinicians with a list of alerts, both for out-of-range PSA and for PROM values. Clinicians can drill down into individual patient profiles and view their PSA values over time. They are also able to view all submitted PROM values and are alerted to outlier trends that require clinical intervention. Figure 2 presents (a) the graphical view of PSA results; (b) Ned’s Notes; and (c) the EPIC-26 survey.

### Data Collection

#### Adoption of Ned

Our decision to study the adoption of Ned by patients, caregivers, and clinicians was informed by previous pivotal evaluative work on the adoption, nonadoption, and abandonment of a personal electronic health record in the United Kingdom. Greenhalgh et al’s [41] focus on adoption as a primary study outcome revealed a complex and dynamic narrative between patients and health care “experts:” there is a discrepancy between the expectations placed on patients to adopt health technology perceived as beneficial to them, and the reality of what patients actually perceive as beneficial for their own health and well-being [41]. We have therefore selected adoption as a core outcome for our own study and seek to identify the realist context-mechanism-outcome configurations to explain how Ned is adopted (or not) by its users. We define adoption as the active translation of intention into measurable action; this is operationalized as the number of patients, caregivers, and clinicians who are invited to open a Ned account and the consequent number of Ned accounts created.

#### Acceptability of Ned

To achieve effective health promotion using health technology, it is essential that patients perceive a given treatment to be an acceptable and welcome addition to their care [42]. The acceptance of technology has been widely studied, with efforts made in recent years to contextualize this understanding within the health care domain [43]. We define acceptability here as the behavioral intention to use a technology, leading to actual use and consequent use behaviors. For this work, we have chosen to frame our measurement of acceptability through adapting and operationalizing the unified theory of acceptance and use of technology (UTAUT) model [42] into a quantitative questionnaire (Multimedia Appendix 1). The UTAUT is a theoretical model and instrument used to assess the likelihood of user acceptance for a new technology. It has been extensively used to evaluate the factors affecting the acceptance and use of new health technologies [44], including in an evaluation of patient acceptance of an automated text messaging system for improved prostate cancer screening adherence [45]. While the original model identifies 7 predictor constructs for behavioral intention (ie, performance expectancy, effort expectancy, social influence, facilitating conditions, hedonic motivation, price value, and habit), we felt it appropriate to modify the model to better align with the context in which Ned will be implemented. We have therefore removed 2 constructs from the overall model, hedonic motivation and price value, and have also added baseline belief in treatment credibility and outcome expectancy as a predictor variable for both behavioral intention and use behavior.

Our justifications for these decisions are as follows: hedonic motivation, defined as enjoyment while using technology, is not a relevant construct to evaluate Ned given that the app was designed to deliver PSA results, which are a source of anxiety for many prostate cancer survivors [9]. Previous research on patient portal acceptance using the UTAUT model has also recommended the removal of this construct, with the rationale that health technology primarily designed to deliver condition-specific information is intrinsically driven by the presence of a health *problem* —something that does not promote enjoyment [46]. Price value was also not an applicable construct for Ned, since patients will be receiving the app for free and will therefore be unable to comment on its value for money. Finally, we are asking patients to rate their belief in the treatment credibility and outcome expectancy of Ned to improve their survivorship care [47]. The degree to which patients initially believe that a given treatment will benefit their health and well-being has been shown to strongly affect treatment outcomes for chronic conditions [48]. We posit that a patient’s initial belief in Ned will correlate with their ultimate acceptance of the app for survivorship care.

#### Effectiveness of Ned

While a substantial amount of research has been done to address the functional impairments caused by prostate cancer treatments, less emphasis has been placed on alleviating the psychological and emotional barriers faced by survivors throughout their survivorship experience. There is recognition that, while overall satisfaction with prostate cancer follow-up care is high, the presence of problematic treatment-related side effects is associated with higher psychological morbidity, poorer self-efficacy, greater unmet needs, and poorer overall health status [49]. Strategies for identifying those men with ongoing problems, alongside new interventions and models of care tailored to individual needs, are needed to improve quality of life. Novel solutions have been devised, notably the successful implementation of a real-time dashboard platform to integrate prostate cancer-specific PROMs into clinical settings that resulted in higher patient quality of life and satisfaction with care [10]. We are encouraged by this work and seek to explore whether our own technology, which combines the collection of PROMs with the provision of PSA values, can achieve similar outcomes. To establish the degree to which Ned can significantly improve how prostate cancer survivors experience their care, we chose to investigate the app’s impact on 5 specific clinical outcomes: health-related quality of life, satisfaction with cancer care, unmet needs, self-efficacy, and prostate cancer-related level of anxiety. We have operationalized our outcomes using a collection of existing validated questionnaires that have been widely used in prostate cancer survivorship research to standardize our work and facilitate future meta-analyses [50]. We have also selected a data collection schedule matching that of the largest prostate cancer survivorship study ever conducted to enable the comparison of any generated data trends with existing population baseline data [8]. In addition to collecting outcomes data, we will also review patient charts to collect the following clinical variables: Gleason score, cancer stage, treatment group (ie, active surveillance, postsurgery, castration resistant, postradiation, postsalvage radiation, and hormone sensitive), and comorbidities. Table 2 lists the data collection schedule for all outcome measures.

**Table 2:** Data collection schedule for outcomes measures.

Measure	Data collection method	n	Baseline	2 months	6 months	12 months
Use data	Log data analytics software	610	Throughout study duration			
Demographic data	Web-based questionnaire	400	x			
Clinical data	Chart review	400	x			
Credibility/Expectancy questionnaire	Web-based questionnaire	400	x			
Expanded Prostate Cancer Composite	Web-based questionnaire	400	x	x	x	x
Prostate Cancer-Related Quality of Life Scales	Web-based questionnaire	400	x			
Service Satisfaction Scale for Cancer Care	Web-based questionnaire	400	x	x	x	x
Supportive Care Needs Survey	Web-based questionnaire	400	x	x	x	x
Self-Efficacy for Managing Chronic Disease Scale	Web-based questionnaire	400	x	x	x	x
Memorial Anxiety Scale for Prostate Cancer	Web-based questionnaire	400	x	x	x	x
Modified UTAUT^a^ survey	Web-based questionnaire	400				x
Patient qualitative interviews	(1) Semistructured live interview (2) Qualitative observation of app use	20				x
Caregiver qualitative interviews	Semistructured live interview	10				x
Clinician System and Use Assessment Survey	Web-based questionnaire	10				x

^a^UTAUT: unified theory of acceptance and use of technology.

#### Health-Related Quality of Life

We will assess prostate cancer-specific quality of life and functional recovery after treatment using the EPIC-26 [39]. The EPIC-26 is a reliable and valid scale with 26 items assessing 5 health domains: urinary continence, urinary irritation, sexual function, bowel function, and hormonal expression. It is scored on a summary scale from 0 to 100, with higher scores corresponding to higher health states. We will also use the Prostate Cancer-Related Quality of Life Scales [51] to complement the EPIC-26, as the scales are designed with a stronger focus on patient perception and derived meaning from treatment outcomes. Each individual scale contains between 2 and 8 items and is rated on a 5-point Likert scale. We will specifically be using the following 6 scales: Health Worry, PSA Concern, Cancer Control, Informed Decision, Regret, and Outlook.

#### Satisfaction with Cancer Care

We will assess patients’ satisfaction with their treatment outcomes using a cancer care-specific adaptation of the Service Satisfaction Scale [52,53]. This scale is often used in combination with the EPIC-26 to capture the relationship between quality of life and satisfaction with outcomes [8]. It consists of 16 items and measures several aspects of satisfaction, including satisfaction with outcomes, provider manner and skill, health information, and access. Responses are scored and converted to a scale from 0 to 100, with higher scores indicating higher levels of patient satisfaction.

#### Unmet Needs

We will use the 34-item Supportive Care Needs Survey Short Form (SCNS-SF34) [54] to assess a patient’s current level of need across 5 domains: psychological, health system and information, physical and daily activity, patient care, and support and sexuality. This validated instrument has been previously used in prostate cancer survivorship studies, where it led to the determination that the most prevalent unmet needs are related to sexual issues, concerns about significant others, and anxieties around the possibility of recurrence [9]. We will be using a modified version of the SCNS-SF34 with a simplified response format, which was validated for use with prostate cancer survivors [55].

#### Self-Efficacy

The Self-Efficacy for Managing Chronic Disease Scale [54] was developed to measure self-efficacy in people with chronic conditions. It has been previously used in a cancer patient population, and has also been adapted to assess cancer-specific self-management behaviors. We will therefore be using the adapted Cancer Survivors Self-Efficacy Scale for this research [49]. Patients will rate their confidence to perform 6 self-management behaviors on a scale of 1 to 10. A mean score will then be calculated, with a higher value indicating greater self-efficacy. Previous investigations into prostate cancer survivor self-efficacy have been positive; patients reported being generally confident with their ability to keep their symptoms or health problems from interfering with their lives. In relation to cancer specifically, patients were also generally confident they could access information and support, deal with problems the cancer may have caused, and contact their clinicians with any problems. We are interested in exploring whether our patient population will share a similar self-efficacy status.

#### Prostate Cancer-Related Level of Anxiety

We will assess the psychological difficulties faced by prostate cancer survivors using the Memorial Anxiety Scale for Prostate Cancer (MAX-CP) [56], which has been validated to measure anxiety in men with prostate cancer receiving ambulatory care. This 18-item scale consists of 3 subscales measuring anxiety related to prostate cancer, fear of recurrence, and PSA-related anxiety. In the original validation study, it was anticipated that patients with rising PSA levels would display more PSA-related anxiety; however, this hypothesis received only limited support and correlated modestly with changes in MAX-CP scores. We would like to further explore this correlation in our own patient population to better understand the sensitivity of PSA-related changes to anxiety status.

#### Patient Experience of Ned

In addition to exploring the acceptability of Ned by patients, we will also ask them about their experience using the app to capture emerging themes of how it translates into a real-world setting. We will conduct semistructured interviews with 20 patients at study conclusion, alongside a qualitative observation session where we will ask patients to perform a series of tasks in Ned while observed by an evaluator. The contents of this interview will be modeled after the Greenhalgh interview framework [41] (Multimedia Appendix 2). This will help us to determine the design-translation gap and improve the app’s workflow. The research team will recruit interviewees through snowball sampling all patients who complete their 12-month study assessment and consent to being interviewed. This will mean that patients who are enrolled earlier will have a greater chance of being interviewed poststudy about their experience with Ned. Our justification for this decision is proactive in nature: we want to understand how patients experience Ned as early as possible so that we can address any reported concerns or difficulties with the app and improve it for the remaining participants in the study. This will ultimately ensure that the least number of patients possible will be exposed to an unfavorable version of Ned.

#### Caregiver Experience of Ned

The experience of prostate cancer survivorship is often a shared one, with the caregivers of survivors taking on the responsibility of advocating for greater survivorship care [57]. Caregivers should be fully integrated into the circle of care and have access to the same information as patients do if they are expected to effectively advocate on their behalf. We believe that for Ned to have a meaningful impact on the survivorship experience, the app must enable caregivers to easily access their partner’s health data to create a full record of care. Ned must also be a seamless and facilitating addition to the tasks of care. As caregivers advocate for their partners’ health needs, a fluid transfer of data between patient, caregiver, and clinician that can be referenced and acted upon during point-of-care interactions may help to improve overall survivorship care. With Ned as a resource, caregivers may be able to prevent the loss of laboratory results and gain the opportunity for evidence-based discussions of symptoms where clinical interactions are not subject to recall bias and nerves. We will explore how caregivers perceive using Ned to support their partners through conducting a semistructured interview with 10 caregivers, the contents of which will be modeled after the Greenhalgh interview framework [41] and the modified EPIC-Partner framework [8,57] (Multimedia Appendix 3).

#### Clinician Experience of Ned

Future scalability and sustainability efforts for Ned will depend on how clinicians perceive the app and their willingness to champion its use in hospitals and homes. We will use the Canada Health Infoway System and Use Assessment Survey to assess clinician adoption, use, and satisfaction with Ned alongside information and system quality [58]. We have modified the original survey to elicit Ned *-* specific insights and will be delivering the survey to 10 clinicians as a Web-based questionnaire to maximize convenience and ease of survey completion.

### Sample Size

The appropriate sample size for single-case research depends on the specific research question being investigated and cannot be calculated in the same way as group designs such as the randomized controlled trial. In his seminal writing on case study designs, Yin recommends abandoning traditional sampling logic in favor of reflecting on the number of replications that are desired to maximize both certainty of research findings and external validity [59]. However, we recognize that our study design diverges from traditional case study methodology in nesting a within-group pre-post comparison of health outcomes aimed at identifying possible parameters of clinical change from baseline to study conclusion. We have therefore diverged from Yin’s recommendations and performed a sample size calculation powered to detect a minimal clinically important difference (MCID) of 4 with a standard deviation of 22.25 on the EPIC-26. We selected the most conservative MCID and the median standard deviation from the range of validated values (MCID range 4-12, SD range 12.6-31.9), which have been widely used in prostate cancer survivorship research [60]. With 90% power, an effect size of Cohen *d*=0.16, and a 2-sided significance level of 5%, a minimum of 317 participants are required to detect this MCID. We anticipate a dropout rate of 20%, bringing our total sample size to 400 prostate cancer survivors for recruitment. We performed this calculation using the G*Power 3.1 software [61]. We will actively recruit the 400 patients required to power our within-group pre-post comparison of health outcomes, but we do not have a predetermined sample size for caregivers and clinicians. We anticipate that 10 clinicians will accept the invitation to join the Ned study and 50% of patients will share Ned with their caregivers. This will result in a total study sample size of 610 participants, composed of 400 patients, 200 caregivers, and 10 clinicians.

### Data Analysis

#### Qualitative Interview Data

The audio recordings for all 30 interviews will be transcribed verbatim. Two members of our research team, (ie, a researcher and research analyst) will first separately code data from 3 patient and 3 caregiver interviews, and record insights and reflections from the data. A conventional qualitative content analysis approach will be used to code qualitative data [62]. Specifically, both researcher and analyst will read through the first interview transcript from beginning to end, similar to reading a novel. Then, they will reread and sort the transcript to identify similar phases, patterns, themes, sequences, and additional important features [63]. These words will become preliminary codes and organized into a coding scheme for use on the remaining 2 interviews. The researcher and analyst together will compare codes and either combine or add new codes. These generalizations will be examined in light of existing knowledge, and representative descriptive texts will be generated. These texts will inform a study codebook, which will then be used to code the remaining 17 patient and 7 caregiver interviews. This codebook will also inform the content analysis process, where descriptive texts will be divided into appropriate thematic categories [64]. We will use the case study analytic techniques of pattern matching and explanation building to build a valid realist program theory [59]. All interview transcripts will be analyzed and coded using NVivo version 11 (QSR International).

#### Quantitative Survey Data

Descriptive statistics will first be conducted on all variables to identify methodological data trends and parameters. We will analyze differences in baseline demographic and clinical variables using Pearson chi-square and nonparametric Wilcoxon rank sum tests. Given that we will be asking the same patients to complete repeated outcome measures across 4 time periods, we will account for autocorrelation effects through the use of linear generalized estimating equations, which are a multivariate analog of linear regression for longitudinal data and recommended for use in longitudinal repeated-measures data analyses [65,66]. Our data analysis strategy to answer the following questions is as follows:

(1) What is the effect of Ned on health-related outcomes over time? To assess the effect of Ned on quality of life, satisfaction with cancer care, unmet needs, self-efficacy, and prostate cancer-related level of anxiety domains, we will use generalized estimating equation models containing indicators for each study time point to assess whether the average study assessment scores at 2 months, 6 months, and 12 months were significantly different from baseline values.

(2) Is there a relationship between patient engagement with Ned and changes in health-related outcomes over time? We will explore whether patients who engage with the content and functionality of the app experience improvements in their health and well-being by building linear regression models for use and outcomes data.

(3) Is there a relationship between health-related outcomes? To understand whether changes in health-related outcomes share a similar direction and magnitude, we will perform Pearson correlation analyses to test for a correlational relationship between outcome variables.

(4) Do demographic and clinical variables affect changes in health-related outcomes? We will first conduct independent *t* tests and 1-way independent analyses of variance to determine whether there were differences in health-related outcomes for all categorical demographic and clinical variables, and Pearson correlation analyses for all continuous variables. We will then perform multiple linear regression analyses with health-related outcomes as dependent variables and all significant predictor variables from our preliminary analyses as independent variables.

We will triangulate both quantitative and qualitative data to develop the realist context-mechanism-outcome pattern configurations required to generate a program theory that explains how Ned is experienced by patients, caregivers, and clinicians.

## Results

We recruited our first survivor in October 2017 and expect to reach our sample size requirements by March 2018. The anticipated completion date for this work is May 2019, and we aim to disseminate study findings through peer-reviewed publications and presentations starting September 2019.

## Discussion

While there is mounting evidence to support the provision of digital prostate cancer survivorship care [18-22], there has not yet, to our knowledge, been an integrated effort to combine care strategies into a single platform for access by patients and their circle of care. Clinical researchers have not yet developed or comprehensively investigated digital interventions capable of eliciting the personal and medical context required to provide appropriate survivorship care. Further, with few exceptions [21,67-69], most existing studies for evaluating digital prostate cancer survivorship resources have been primarily formative in scope and have focused on nascent Web-based technologies [20,70-73]. This leaves a gap in the literature for evaluative research to build on this foundation of knowledge and advance a realist understanding of how to adopt and implement digital prostate cancer survivorship solutions across diverse contexts.

We have endeavored to design an evaluation that can respectfully explore the nuanced relationship between prostate cancer survivors and a technology designed to improve their survivorship care. To our knowledge, this is the first realist case study evaluation of an mHealth platform for prostate cancer survivorship care. The case study design employed for this work is supported by a mixed-methods approach to data collection [59], a strong theoretical grounding in realist principles [33], a pragmatic data collection schedule that aligns with existing implementation timelines [74], and a shared focus between observational and experimental investigation [75]. We have made efforts to select study instruments that are thoughtful in their wording and specific to prostate cancer survivorship, so that patients will understand the relevance of what is being asked of them. Our inclusion of caregiver interviews as both a distinct exploration into the health and well-being of partners and also a complement to the patient survivorship experience is more comprehensive than focusing on survivors alone.

It is acknowledged that most men with a diagnosis of prostate cancer will die *with* it, not *of* it [76-78]; therefore, prostate cancer is a chronic condition that requires effective management. We posit that Ned has the potential to support this management through the systematic monitoring of outcomes and may contribute to a measurable and meaningful shift in health and well-being. The lives of prostate cancer survivors are marked by unresolved needs that are not often addressed through their interactions with their circle of care [79]. We are hopeful that by providing survivors, caregivers, and clinicians with a foundation of shared health information, Ned will initiate informed conversations around the provision and acceptance of empathetic care.

Prostate cancer survivorship care will be at the forefront of health care resource allocation by 2030. Prostate cancer survivors are set to increase in number and longevity, heightening the need for integrated survivorship solutions to provide them with optimal and durable outcomes. We believe that Ned marks the proactive start of a shift in prostate cancer care innovation. We aim for this research to explore the app’s potential to empower the survivorship experience and inform a new era of prostate cancer survivorship care.

## Appendix

Multimedia Appendix 1 Ned patient acceptance and use of information technology survey.

Multimedia Appendix 2 Ned patient interview questions.

Multimedia Appendix 3 Ned caregiver interview questions.

## Abbreviations

EPIC-26: Expanded Prostate Cancer Index Composite
MAX-CP: Memorial Anxiety Scale for Prostate Cancer
MCID: minimal clinically important difference
OLIS: Ontario Laboratories Information System
PROM: patient-reported outcome measure
PSA: prostate-specific antigen
REB: research ethics board
SC: study coordinator
SCNS-SF34: 34-item Supportive Care Needs Survey Short Form
THP: Trillium Health Partners
UTAUT: unified theory of acceptance and use of technology
