# Supplementary material for: Adoption, Acceptability, and Effectiveness of a Mobile Health App for Personalized Prostate Cancer Survivorship Care: Protocol for a Realist Case Study of the Ned App
Source: JMIR Res Protoc. 2017 Oct 12;6(10):e197. doi: 10.2196/resprot.8051 (PMC5658643; doi:10.2196/resprot.8051)
Supplement: Multimedia Appendix 1 [file resprot_v6i10e197_app1.pdf]

Appendix 1. Ned Patient Acceptance and Use of Information Technology Survey

The following statements are meant to assess your acceptance and use of Ned. Please select the number that matches your agreement with the statement.

		Strongly disagree<br>1	2	2	4	5	6	Strongly agree<br>7
1.	I found Ned useful in my daily life.							
2.	Using Ned helped me accomplish things more quickly.							
3.	Using Ned increased my productivity.							
4.	Learning how to use Ned was easy for me.							
5.	My interactions with Ned were clear and understandable.							
6.	I found Ned easy to use.							
7.	It was easy for me to become skilful at using Ned.							
8.	People who are important to me think that I should use Ned.							
9.	People who influence my behavior think that I should use Ned.							
10.	People whose opinions that I value prefer that I use Ned.							
11.	I have the resources necessary to use Ned.							
12.	I have the knowledge necessary to use Ned.							
13.	Ned is compatible with other technologies I use.							
14.	I can get help from others when I have difficulties using Ned.							
15.	The use of Ned has become a habit for me.							
16.	I am addicted to using Ned.							
17.	I must use Ned.							
18.	I intend to continue using Ned in the future.							
19.	I will always try to use Ned in my daily life.							
20.	I plan to continue to use Ned frequently.							

Thank you very much for taking the time to answer these questions!
