# Supplementary material for: Adoption, Acceptability, and Effectiveness of a Mobile Health App for Personalized Prostate Cancer Survivorship Care: Protocol for a Realist Case Study of the Ned App
Source: JMIR Res Protoc. 2017 Oct 12;6(10):e197. doi: 10.2196/resprot.8051 (PMC5658643; doi:10.2196/resprot.8051)
Supplement: Multimedia Appendix 2 [file resprot_v6i10e197_app2.pdf]

Adoption, acceptability and effectiveness of a mobile health application for personalised prostate cancer survivorship care: a realist case study of *Ned*.

Appendix 2. Ned Patient Interview Questions

The prompt sheet was adapted flexibly depending on points raised by the interviewee.

1. Tell me a little bit about yourself! What makes you who you are?

2. How would you rate your health literacy or knowledge of health issues?
    a. Do you read up on health news, either specific to your health or just in general?
    b. Do you feel like you have good knowledge of your own health?

3. How would you rate your IT or technology literacy?
    a. Do you engage with a lot of technology in your everyday life? Examples?

4. Tell me about your use of smartphones. Do you use any apps on your smartphone?
    a. *Explore use of smartphone apps, such as internet banking apps, shopping apps like Amazon, communication apps like WhatsApp, social media apps like Instagram.*

5. Are you aware of any health information apps or mobile health apps other than Ned?
    a. Do you use any mHealth apps?
    b. Has this changed since you started using Ned?

6. If you're comfortable doing so, can you tell me a little bit about your experience with prostate cancer survivorship?
    a. What would you say is the frequency and quality of your interactions with the healthcare system, so for example, the number of times you go to see your doctor or go to the clinic, and how you experience those visits?

7. How and how often did you communicate with your doctor before using Ned?
    a. Has this changed since you've started using Ned? If so, how?

8. How was Ned introduced to you by your clinician?
    a. How were its potential benefits and drawbacks described to you?

9. How did you set up Ned on your smartphone?
    a. Did anyone help you? How easy or hard was it, and did you experience any specific issues?

10. What were your expectations of Ned before using it?

11. Tell me about your experience using Ned.
    a. If you haven't used Ned, I'm also just as interested in understanding why not.
    b. What features of the app do you use the most? What features do you use the least?
    c. What features of the app do you find most useful? What features do you find least useful?
    d. Can you give me some examples of how you would use Ned in your everyday life?

12. Has Ned had any effect on the way you experience your prostate cancer survivorship?
    a. If so, how?

13. Has Ned had any effect on the way you communicate with your clinician?
    a. Do you see your clinician more or less often now that you use Ned?
    b. Have your interactions with the healthcare system changed?

14. Has Ned had any effect on the way you communicate with your carer?

15. Has Ned had any effect on the way you report any symptoms or health concerns to your circle of care?

16. If you could, what would you change about Ned, and why?

17. How would you feel if Ned was no longer offered to you?

18. Would you recommend Ned to someone going through the same prostate cancer survivorship experience as yourself, and why?

19. Do you have any final thoughts you wish to share with me?

Now we are going to move onto the part of this interview where we ask all our patients to show us how they have been using Ned for the past year. The reason why we are doing this is to see whether Ned works as intended and is being used the way we designed for it to be used. I just want to be clear in saying that we are testing Ned, not you! There is no right or wrong way to use Ned, and we just want to learn from how you personally use the application. As you use Ned, I'm going to ask you as much as possible to try to think out loud: to say what you're looking at, what you're trying to do, and what you're thinking. This will be a big help to us.

You may have noticed the video camera. With your permission, we're going to video record what happens on the screen and our conversation. The recording will only be used to help us figure out how to improve the site, and it won't be seen by anyone except the people working on this project. And it helps me, because I don't have to take as many notes.

We are going to use this phone with a pre-populated version of Ned instead of your own phone so that I don't see any of your personal data. I am now going to ask you a series of questions about some specific features of Ned.

Task 1
Can you show me how you usually complete the EPIC-26 and FACT-P surveys in Ned?
Can you share with me what you did or did not like about this Survey feature?

Task 2
Please show me what you would do to look at an old EPIC-26 score that you completed 6 months ago?
Can you share with me how you found these survey graphs to look and work?

Task 3
Can you show me what you would do once you received a notification that you had a new PSA result?
How did you find this process? Would you have changed anything about it?

Task 4
Please walk me through the process of how you would add a caregiver to your account.
Can you share with me your thoughts on this particular feature of the app? Did you ever use this feature? What were your reasons for using/not using this feature?

Task 5
Finally, can you please walk me through one of your typical sessions with Ned? Let's start from the beginning – what do you usually do right after you enter your password and go into the home screen?
