# Supplementary material for: Adoption, Acceptability, and Effectiveness of a Mobile Health App for Personalized Prostate Cancer Survivorship Care: Protocol for a Realist Case Study of the Ned App
Source: JMIR Res Protoc. 2017 Oct 12;6(10):e197. doi: 10.2196/resprot.8051 (PMC5658643; doi:10.2196/resprot.8051)
Supplement: Multimedia Appendix 3 [file resprot_v6i10e197_app3.pdf]

Adoption, acceptability and effectiveness of a mobile health application for personalised prostate cancer survivorship care: a realist case study of Ned.

Appendix 3. Ned Caregiver Interview Questions

The prompt sheet will be flexibly adapted depending on points raised by the interviewee.

1. Tell me a little bit about yourself! What makes you who you are?
2. If you're comfortable doing so, can you tell me a little bit about your experience as a caregiver for a partner who is a prostate cancer survivor?
   a. What would you say is the frequency and quality of your interactions with the healthcare system, so for example, the number of times you accompany your partner to go to see their doctor or go to the clinic
   b. What were your experiences with these visits like?
3. How and how often did you communicate with your partner's doctor before using Ned?
   a. Has this changed since you've started using Ned? If so, how?
4. How was Ned introduced to you, and how were its potential benefits and drawbacks described to you by your partner?
5. How did you set up Ned on your smartphone?
   a. Did anyone help you? How easy or hard was it, and did you experience any specific issues?
6. Tell me about your experience using Ned.
   a. If you haven't used Ned, I'm also just as interested in understanding why not.
7. Has Ned changed the way you communicate with your partner?
8. Has Ned changed the way you communicate with your partner's doctor?
9. As a caregiver, have your interactions with the healthcare system changed?
10. Has Ned changed the way you experience your partner's prostate cancer survivorship?
    a. If so, how?
11. If you could, what would you change about Ned, and why?
12. How would you feel if Ned was no longer offered to you and your partner?
13. Would you recommend Ned to another caregiver going through the same prostate cancer survivorship experience as yourself?
14. Do you have any final thoughts you want to share with me?

Ned Expanded Prostate Cancer Index Composite–Partner Survey

1. How much has your husband's or partner's urinary incontinence, such as urinary leakage or loss of urinary control been a problem for you during the last four weeks?
2. How much has your husband's or partner's urinary irritation or blockage, such as frequent urination, pain or burning with urination, urinary urgency, waking up to urinate, blood in the urine, or related difficulties in passing his urine, been a problem for you during the last four weeks?
3. How much has your husband's or partner's overall urinary function, such as urinary leakage, incontinence, frequent urination, urinary urgency, urinary burning, urinary bleeding, waking up to urinate, or other urinary difficulties been a problem for you during the last four weeks?
4. How much has your husband's or partner's bowel habits, such as rectal urgency, frequent bowel movements, leakage of stool, bloody stool, or painful bowel movements, been a problem for you during the last four weeks?
5. How much has your husband's or partner's sexual function such as his degree of sexual desire, the frequency and quality of his erections, or the level of sexual activity, been a problem for you during the last 4 weeks?
6. How much has your husband's or partner's hormone function and vitality, such as lack of energy, hot flashes, breast tenderness, weight gain, or mood changes, been a problem for you during the last 4 weeks?

Response options:

1	2	3	4	5
No problem	Very small problem	Small problem	Moderate problem	Big problem
